# Loneliness, social isolation and social relationships: what are we measuring? A novel framework for classifying and comparing tools

**DOI:** 10.1136/bmjopen-2015-010799

**Published:** 2016-04-18

**Authors:** Nicole K Valtorta, Mona Kanaan, Simon Gilbody, Barbara Hanratty

**Affiliations:** 1 Department of Health Sciences, University of York, Heslington, York, UK; 2 Institute of Health and Society, Newcastle University, Newcastle upon Tyne, UK

**Keywords:** social relationships, social epidemiology, loneliness

## Abstract

**Objectives:**

We present a novel way of classifying and comparing measures of social relationships to help readers interpret the growing literature on loneliness and social isolation and to provide researchers with a starting point to guide their choice of measuring tool.

**Methods:**

Measures of social relationships used in epidemiological studies were identified from two systematic reviews—one review on the association between social relationships and health and social care service use, and a second review on the association between social relationships and health. Questions from each measure were retrieved and tabulated to derive a classification of social relationship measures.

**Results:**

We present a classification of measures according to two dimensions: (1) whether instruments cover structural or functional aspects of social relationships and (2) the degree of subjectivity asked of respondents. We explain how this classification can be used to clarify the remit of the many questionnaires used in the literature and to compare them.

**Conclusions:**

Different dimensions of social relationships are likely to have different implications for health. Our classification of social relationship measures transcends disciplinary and conceptual boundaries, allowing researchers to compare tools that developed from different theoretical perspectives. Careful choice of measures is essential to further our understanding of the links between social relationships and health, to identify people in need of help and to design appropriate prevention and intervention strategies.

**Strengths and limitations of this study**

- We systematically searched for tools measuring social relationships, following the Centre for Reviews and Dissemination guidelines.
- We classified measures in a way that transcends disciplinary and conceptual boundaries, allowing us to compare tools developed from different theoretical perspectives.
- Besides providing an easy interpretation of existing research for researchers, policymakers and practitioners, the classification we present can help guide researchers' choice of measure in future studies.
- Other factors that need to be taken into account when choosing tools, and that are not covered in this paper, include psychometrics, study population and study hypothesis.

## Introduction

Social relationships ‘exist between two people when each person influences the other's thoughts, feelings, and or behaviour, [i.e.] when people are at least minimally interdependent’.^1^ Their influence on health is attracting growing interest from policymakers and practitioners, amidst concern about the well-being of certain groups, in particular older adults, in increasingly fragmented industrialised societies.^2–4^ We know from reviews of the research evidence that people with weaker social relationships are at greater risk of premature mortality.^5^ What we do not know is whether some aspects of relationships (eg, their quality or quantity; subjectively vs objectively assessed availability) are more problematic than others, and for whom.

One of the main reasons why we know little about the comparative effects of different social relationship dimensions is the inconsistent use of terminology. In the absence of a comprehensive framework, investigators from a range of disciplines, including sociology, psychology, demography and epidemiology, have suggested definitions of concepts that cannot always easily be reconciled. For example, House and Khan proposed to distinguish between two dimensions of social relationships: social network and social support.^6^ They defined social network as the structural dimension of social relationships, encompassing aspects such as the density, duration, dispersion, reciprocity and homogeneity of relationships. Social support was defined as the functional aspect of relationships (ie, covering aspects such as the provision or receipt of information, instrumental help, emotional support or advice). In contrast, O'Reilly suggested instead that social network be used as the main concept, with social support as a subsidiary concept covering the qualitative and behavioural aspects of the social network.^7^

Approaches to operationalising tools have been similarly heterogeneous, so it is often unclear how different measurement tools differ or overlap, making comparison difficult. This raises a number of questions: How do researchers choose their measure? Are these measures relevant to the population under study? Do questionnaires capture what they purport to measure? In this study, we propose a new way of classifying measures of social relationships. Our aim is to provide a transparent and accessible way of reviewing tools to help readers understand and interpret the existing evidence.

### Rationale for developing a classification of measurement tools

There are many instruments available for assessing different aspects of social relationships: the Berkman-Syme Social Network Index,^8^ the Lubben Social Network Scale,^9^ the de Jong Gierveld Loneliness Scale,^10^ the UCLA Loneliness Scale^11^ and the Interview Schedule for Social Interaction,^12^ for example. Exactly what these tools are designed to measure is often unclear. Researchers have tended to use terms including social integration, social ties or social isolation loosely and interchangeably, so that labels such as ‘measure of social support’ or ‘social interaction scale’ are not reliable indicators. For example, in an article reporting results from the Prospective Epidemiological Study of Myocardial Infarction, we read that ‘social support’ was measured using the Berkman-Syme Social Network Index.^13^ In a systematic review of observational studies on psychosocial factors and coronary heart disease, ‘social support’ was understood to encompass a range of situations and measurement tools, including ‘high love and support from wife’, ‘social network index’ and ‘social isolation’.^14^

An important reason for clarifying the literature is that different domains of social relationships might have different implications for health. Unfortunately, most epidemiological studies focus on only one measure of social relationships, precluding direct comparisons. Evidence from the few studies that do include measures of objective as well as subjective aspects of social relationships suggests that the two dimensions are weakly correlated, and that they have independent effects on health-related outcomes.^15–17^ A single approach to measuring social relationships is therefore unlikely to be appropriate for all purposes, and investigators need to choose measurement tools carefully, basing their choice on clear hypotheses of how and why social relationships might influence particular health outcomes.^18^

To overcome the lack of conceptual clarity in the literature and to help researchers choose measurement tools tailored to their needs and objectives, we propose a way of classifying instruments that allows comparison across disciplinary boundaries. Our classification builds upon a distinction frequently referred to in the literature, the difference between the functional (qualitative) and structural (quantitative) aspects of social relationships,^19^ and takes into account a second, important, dimension: the way in which questionnaire items are phrased, which informs us about the degree of subjectivity asked of respondents.

## Methods

We developed a classification in two stages. First, we systematically searched for studies on the association between social relationships and health and social care service use among adults aged 65 and over. Searches were tailored to eight electronic databases (MEDLINE, EMBASE, Scopus, Web of Science, CINAHL Plus, the Cochrane Library, the Centre for Reviews and Dissemination database and PsycINFO) using a combination of index headings (eg, ‘Loneliness’, ‘Social isolation’, ‘Social support’) and free text terms (see online supplementary appendix 1 for the search strategy used in MEDLINE), and were last updated in October 2015. The reference lists of relevant studies were screened for further eligible records. The 32 205 records identified were screened by two researchers who selected studies which included a measure of the quantity and/or quality of individuals' social relationships. We applied no study design, language, publication type or date restrictions. For each study, we retrieved the questions used to assess social relationships and grouped them according to how they were formulated. Through this process, we identified two ways in which questions differed: (1) whether they were asking about the structure or the function of social relationships, and (2) whether respondents were being asked to report on past and present contact with others; availability of relationships as they perceive it; adequacy of their relationships; and feelings relating to social relationships.

10.1136/bmjopen-2015-010799.supp1 Supplementary appendix 1

In the second phase, we tested whether a framework based on these two dimensions could be used to classify the measures used in studies on social relationships and cardiovascular disease. To identify these studies, we searched 16 electronic databases (MEDLINE, EMBASE, CINAHL Plus, PsycINFO, ASSIA, Web of Science, the Cochrane Library, Social Policy and Practice, National Database of Ageing Research, OpenGrey, HMIC, ETHOS, NDLTD, NHS Evidence, SCIE and NICE), using a combination of thesaurus and free text terms including loneliness, social isolation, social relationships, social support and social network (search last updated in May 2015; for an example of the full electronic strategy used to search MEDLINE, see online supplementary appendix 2). The titles and abstracts of the 35 925 records identified were independently screened by two researchers, who selected eligible studies based on whether they included a measure of the quality and/or quantity of individuals' social relationships.

10.1136/bmjopen-2015-010799.supp2 Supplementary appendix 2

### Results

Our systematic searches identified 54 instruments (see online supplementary appendix 3 for a full list, including references to the studies in which each tool was used, and references to the original article or report in which the tool was described). The number of questions in each tool ranged from 1 to 32. Taking each question at a time, we considered its content and the way in which it was formulated. This allowed us to develop a classification based on (a) whether the question was about the function or structure of social relationships and (b) the degree of subjectivity which it required from respondents.

10.1136/bmjopen-2015-010799.supp3 Supplementary appendix 3

#### First dimension: structure versus function

Questions that touch on the structure of social relationships seek to find out who people share an interpersonal relationship with, and to assess the linkages between these individuals.^20^ Structural characteristics of social relationships cover the number and type of people with whom a person interacts, the diversity, density and reciprocity of a person's social network, and frequency and duration of contact between individuals. Examples of questions concerned with structure include: ‘Have you ever been married? If so, are you now married, separated, divorced or widowed?’ (Berkman-Syme Social Network Index)^8^ and ‘How many relatives do you see or hear from at least once a month?’ (Lubben Social Network Scale).^9^

Questions on the functional aspects of social relationships target the qualitative and behavioural characteristics of interactions and exchanges between people.^20^ These questions are about the purpose and nature of relationships, with much of the literature focusing on their beneficial functions, in particular receiving and providing social support. This can take the form of emotional help (eg, expressions of love and caring), tangible aid (eg, transport), information or companionship.^21^ While much of the epidemiological literature has focused on social support as the mechanism through which social relationships affect health, we note that other functions are likely to affect health too, notably social influence and engagement, and opportunities for person-to-person contact.^18^ Examples of questions concerned with function include: ‘At present, do you have someone you can share your most private feelings with (confide in) or not?’ (Interview Schedule for Social Interaction)^12^ and ‘How often is there someone available to take you to the doctor if you needed it?’ (MOS Social Support Survey).^22^

#### Second dimension: the degree of subjectivity asked of respondents

All answers to self-report questionnaires involve a degree of subjectivity; nevertheless, when comparing questions on social relationships, we found that the degree of subjectivity expected of respondents varied, based on the way in which items were formulated. In the following section, we describe each of the four different formulations we identified, starting with the more objective questions and progressively moving towards greater subjectivity.

- Items assessing respondents' involvement in social relationships

A first type of question aims to capture people's access to social relationships using a relatively objective approach. These questions often, but not always, ask individuals to quantify their social relationships and require a numerical answer. For example: ‘How many relatives do you see or hear from at least once a month?’ (possible answers: 0, 1, 2, 3 or 4, 5–8 or 9+; Lubben Social Network Scale).^9^ Such questions attempt to gauge the size and range of social relationships in which a person is involved, although we note that answers could be telling us more about individuals' needs rather than access—that is, people might not have engaged in certain social relationships because they did not feel the need to, rather than because they could not.

- Items assessing the availability of social relationships as perceived by respondents

A second way of assessing access to social relationships is to ask people whether such relationships are available to them. For example, in a four-item measure of social isolation used in the Japan Public Health Center-based Prospective Study II, participants were asked: ‘Do you have someone who is supportive of your opinions and actions?’.^23^ Questions are often phrased hypothetically, for example: ‘Is there someone who would give you any help at all if you were sick or disabled, for example, your husband/wife, a member of your family, or a friend?’ (OARS Social Resources Scale).^24^ Such questions do not tell us about whether social relationships are actually available to individuals, but are a measure of availability as perceived by respondents.

- Items assessing the adequacy of social relationships from respondents' perspective

A third type of question asks respondents to report on whether they are satisfied with the quality and/or quantity of their interaction with others. Examples include: How satisfied are you with the kinds of relationships you have with your family and friends? (possible answers: very dissatisfied, somewhat dissatisfied, satisfied; 11-item Duke Social Support Index)^25^; ‘I find my circle of friends and acquaintances too limited’ (possible answers: ‘yes!’ ‘yes’, ‘more or less’, ‘no’ and ‘no!’ or ‘yes’, ‘more or less’ and ‘no’; de Jong Gierveld Loneliness Scale).^10^ Answering such questions requires participants to appraise their social relationships against their expectations.

- Items where respondents are asked about their feelings relating to social relationships

A last type of question focuses on feelings associated with social relationships. For example, in the UCLA Loneliness Scale, respondents are asked whether they ‘feel isolated from others’, ‘feel left out’ or ‘feel completely alone’.^11^ Questions can cover positive and negative feelings, and ask how people feel about the quality as well as the quantity of their relationships.

#### Using the classification to clarify what each questionnaire is measuring

As we developed our classification, it became apparent that while the majority of questionnaires were designed with a total score in mind (ie, no subscales), they often included more than one type of question. In table 1, we list the 54 instruments identified from our systematic searches, and the dimensions they cover. Asterisks indicate that subscales are available for this questionnaire.

**Table 1:** Classification of social relationship measures

Tool used	Number of items	Dimension 1: function vs structure		Dimension 2: degree of subjectivity			
		Structure	Function	Involvement in relationships	Perceived availability	Perceived adequacy	Feelings/emotions
Berkman-Syme Social Network Index*	4	X	X	X			
11-item de Jong Gierveld Loneliness Scale*	11		X		X	X	X
35-item Duke Social Support Index	32	X	X	X	X	X	
11-item Duke Social Support Index	11	X	X	X	X	X	
4-item Duke Social Support Index	4	X	X	X	X		
Duke-UNC Functional Social Support Questionnaire	11	X	X			X	
ENRICHD Social Support Inventory (ESSI)	7	X	X	X	X	X	
Gijón Scale for the elderly's social-family assessment, family and social relationships subscales	10	X		X			
12-item Interpersonal Support Evaluation List (ISEL)	12		X		X		
Interview Measure of Social Relationships	Data not found	X	X	X	X	X	
Litwin Support Network Types	7	X		X			
10-item Lubben Social Network Scale	10	X	X	X	X		
6-item Lubben Social Network Scale	6	X	X	X	X		
Medical Outcomes Study (MOS) Social Support Survey	20		X		X		
Multidimensional Scale of Perceived Social Support (MSPSS)	12		X		X		
Negative Affect Scale	5		X				X
Nottingham Health Profile Social Isolation subscale	5		X		X		X
Older Americans Research and Service Center (OARS) Social Resource Scale	7	X	X	X	X	X	X
Oslo-3 Social Support Scale	3		X		X		
Personal Resource Questionnaire (PRQ2000)	15		X		X	X	X
University of California, Los Angeles (UCLA) Loneliness Scale	20		X		X	X	X
Wenger Support Network Typology	8	X		X			
A measure of social isolation (LaVeist, 1997)	2	X		X			
A measure of social network (Mechakra-Tahiri, 2011)	4	X		X			
A measure of social anchorage (Rennemark, 2009)	4		X				X
Questionnaire on social network (Rodriguez-Artalejo, 2006)	4	X		X			
Question about the number of sources of support (Tennstedt, 1993)	1	X	X	X			
An index of social support (Lai, 2006)	5	X	X	X	X		
A measure of living arrangements and informal care (Crets, 1996)	2	X		X			
A measure of satisfaction with social support (Feld, 1994)	6		X		X	X	
A measure of social integration (Orth-Gomer, 1996)	6	X	X	X	X		
A measure of social isolation (Cloutier-Fischer, 2009)	2	X	X	X	X		
A measure of social network (Reed, 1983)	9	X		X			
A measure of social network (Reed, 1984)	4	X		X			
A measure of social support (Tran, 1997)	5	X		X			
A measure of social support (André-Petersson, 2006)	13		X		X	X	X
A measure of social support (Ikeda, 2008)	4	X	X	X	X		
A measure of social support (Kuper, 2006)	6	X		X	X		
A social network index (Rutledge, 2008)	12	X		X			
Social network type (Coe, 1984)	2	X		X		X	
Social network type—family (Coe, 1985)	2	X		X		X	
Multi-item measures combining questions about frequency of contact with others and participation in activities	2 or more	X		X			
Question(s) about frequency of face-to-face and/or phone contact with family and/or friends and/or neighbours—eg, ‘How many times during the past week did you spend some time with someone who does not live with you?’ (Hyduk, 1996)	1 or more	X		X			
Question(s) about the geographical proximity of family and friends	1	X		X			
Question(s) about the number of close friends or relatives—eg, asking respondents for the ‘number of friends [they] feel close to’ (Lee, 2008)	1 or more	X	X	X			
Question(s) about participation in social activities such as going to the cinema, sport events, church attendance or volunteering—eg, ‘In the past two weeks, did you go to a show or movie, sports event, club meeting, classes or other group event?’ (The Longitudinal Study of Aging, 1992)	1 or more	X					
Question(s) about the perceived availability of emotional, tangible, informational and/or other support—eg, ‘Is there someone who would give you any help at all if you were sick or disabled, for example your husband/wife, a member of your family, or a friend?’ (Barresi, 1987)	1 or more		X		X		
Question(s) about received support—eg, asking participants whether they received assistance during the past month with 7 tasks, including shopping, housework or going to the doctor	1 or more		X				
Question(s) about satisfaction with social relationships and/or participation—eg, asking participants whether they believe their present level of social activities to be adequate	4		X			X	
Question(s) about the size of a person's network—eg, number of friends and relatives outside the household	1 or more	X		X			
Question about time spent alone	1	X		X			
Single-item question about feeling lonely—eg, ‘How often in the last 12 months have you been bothered by loneliness?’	1		X				X

*Subscales available.

#### Using the classification to compare measures

**Figure 1:**
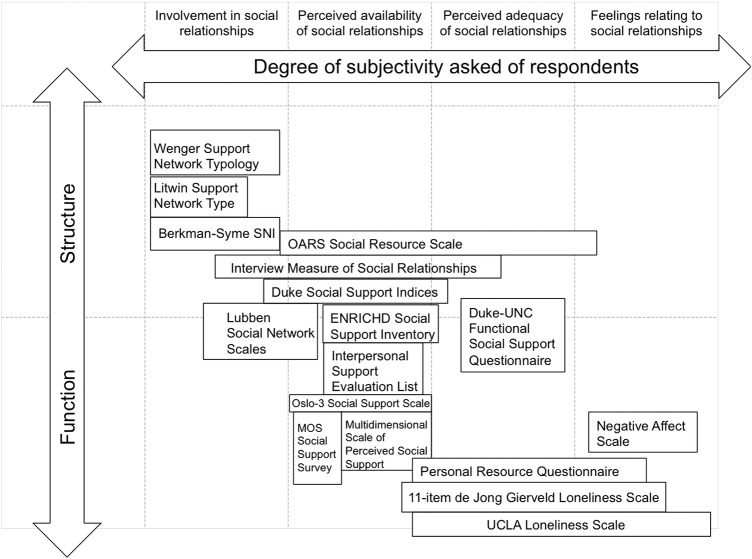
Comparing multi-item questionnaires using a two-dimensional diagram.

Clarifying the remit of each instrument allows us to situate tools in relation to other available measures. In figure 1, we have mapped the multi-item questionnaires developed as stand-alone tools onto a two-dimensional diagram. Questionnaires were placed on the diagram according to whether they contained questions focusing on the structural, functional or both aspects of relationships (vertical axis) and according to the degree of subjectivity asked of respondents (horizontal axis). Where questionnaires contained more than one type of question, for example, the Duke Social Support Index, where participants are asked about their involvement in relationships, as well as to report on the perceived availability and adequacy of relationships, they were mapped accordingly, that is, spanning across these three types of questions. Similarly, where questionnaires included questions about structural as well as functional aspects, they were placed so as to straddle both areas of the diagram (eg, the Lubben social Network Scale, the ENRICHD Social Support Inventory or the Duke-UNC Functional Social Support Questionnaire). For the purpose of clarity, we did not include single-item tools and tools that were developed for specific studies or datasets in our diagram.

Figure 1 allows us to compare and contrast tools. For example, we observe that while they both explicitly target social support, the ENRICHD Social Support Inventory includes questions on the function as well as the structure of relationships, whereas the MOS Social Support Survey focuses on functional aspects only. The diagram also enables us to identify tools with similar foci, and questionnaires that might complement each other. As we might expect, tools explicitly designed for measuring loneliness (eg, the UCLA Loneliness Scale and the de Jong Gierveld Loneliness Scale) tend to be based on more subjective questions, whereas social network indices primarily use more objective measures. Perhaps less intuitively, given that loneliness is commonly defined as referring to the negative feeling associated with people perceiving the quantity and quality of their relationships to be deficient,^26^ we note that tools explicitly designed to measure loneliness tend to focus exclusively on the functional aspects of relationships.

## Conclusions

The classification described in this paper was designed to help readers interpret the existing literature on loneliness and isolation, and to help inform future epidemiological studies on social relationships. One of the ways in which it can be employed is by researchers who intend to review the literature, and who need to define which dimensions of social relationships they are interested in. Rather than rely on inconsistent conceptual terminology, they can use the classification to define the remit of their review (eg, focus on functional or structural dimensions) and identify which measurement tools do and do not fit within their criteria.

Another important way in which the classification can contribute to future research is by helping to guide researchers' choice of measurement tool, since it provides an overview of some of the tools previously used in epidemiological studies and allows investigators to compare instruments developed from different disciplines and theoretical perspectives. Once researchers have compared tools using our framework, they will be in a position to consider other factors of relevance, most importantly, psychometrics (has the tool been validated and shown to be reliable? What of its responsiveness and interpretability?), study population (is the tool adequate for the age group or the cultural context?) and whether the tool captures the most relevant dimensions of social relationships given the investigators' hypotheses about how relationships influence health. Careful choice of measures is essential if we are to further our understanding of how social relationships affect health, and to identify people in need of help. Only by being clear about what is measured can we design appropriate prevention and intervention strategies that target the areas of relationships most problematic for health and well-being.
