# Supplementary material for: Loneliness, social isolation and social relationships: what are we measuring? A novel framework for classifying and comparing tools
Source: BMJ Open. 2016 Apr 18;6(4):e010799. doi: 10.1136/bmjopen-2015-010799 (PMC4838704; doi:10.1136/bmjopen-2015-010799)
Supplement: Supplementary appendix 1 [file bmjopen-2015-010799supp_appendix1.pdf]

Appendix 1 MEDLINE search strategy

Database: Ovid MEDLINE(R) In-Process & Other Non-Indexed Citations and Ovid MEDLINE(R) <1946 to Present>

Searched online 03.04.13

Search Strategy:
----

1. Loneliness/
2. Social isolation/
3. Social distance/
4. Exp Social Environment/
5. lonely.mp.
6. solitude.mp.

7. exp Health Services/
8. exp Patient Care/
9. Home Care Agencies/
10. Home Care Services/
11. Home Health Aides/
12. “social service”.mp.
13. “social care”.mp.
14. utili?ation.mp.

15. 1 or 2 or 3 or 4 or 5 or 6
16. 7 or 8 or 9 or 10 or 11 or 12 or 13 or 14
17. 15 and 16
18. limit 17 to “all aged (65 and over)”
