# Supplementary material for: Loneliness, social isolation and social relationships: what are we measuring? A novel framework for classifying and comparing tools
Source: BMJ Open. 2016 Apr 18;6(4):e010799. doi: 10.1136/bmjopen-2015-010799 (PMC4838704; doi:10.1136/bmjopen-2015-010799)
Supplement: Supplementary appendix 2 [file bmjopen-2015-010799supp_appendix2.pdf]

Appendix 2 MEDLINE search strategy

Database: Ovid MEDLINE(R) In-Process & Other Non-Indexed Citations and Ovid MEDLINE(R) <1946 to Present>

Searched online 10/06/14

Strategy saved as: Loneliness_Medline

Search Strategy:

----

1 loneliness/ (2206)

2 social isolation/ (10940)

3 social alienation/ (1309)

4 social support/ (51329)

5 community networks/ (5430)

6 social distance/ (1444)

7 interpersonal relations/ (55367)

8 Friends/ (2680)

9 psychosocial deprivation/ (1817)

10 Social Participation/ (545)

11 (lonely or loneliness or solitude).ti,ab. (3910)

12 ((social* or societ* or perce* or person*) adj3 (isolation or isolated or alienation or alienated or relation* or detachment or detached or contact or link or tie or ties or support* or network* or participation or activ* or engage* or connect* or disconnect* or cohesion or cohesive or embedded* or vulnerab* or interact*)).ti. (19533)

13 (social wellbeing or social health or social capital).ti. (1205)

14 or/1-13 (134819)

15 exp cohort studies/ (1353453)

16 cohort$.tw. (280225)

17 controlled clinical trial.pt. (88473)

18 epidemiologic methods/ (29786)

19 exp case-control studies/ (662637)

20 (case$ and control$).tw. (331312)

21 or/15-20 (1913522)

22 and/14,21 (15308)
