# Supplementary material for: Loneliness, social isolation and social relationships: what are we measuring? A novel framework for classifying and comparing tools
Source: BMJ Open. 2016 Apr 18;6(4):e010799. doi: 10.1136/bmjopen-2015-010799 (PMC4838704; doi:10.1136/bmjopen-2015-010799)
Supplement: Supplementary appendix 3 [file bmjopen-2015-010799supp_appendix3.pdf]

Appendix 3. List of tools identified, with references

Tool	Studies in which the tool was used
Berkman-Syme Social Network Index [1]	Avendano M, Kawachi I, Van Lenthe F, et al. Socioeconomic status and stroke incidence in the US elderly: the role of risk factors in the EPESE study. *Stroke* 2006;**37**(6):1368-73<br><br>Eng PM, Rimm EB, Fitzmaurice G, Kawachi I. Social ties and change in social ties in relation to subsequent total and cause-specific mortality and coronary heart disease incidence in men. *American Journal of Epidemiology* 2002;**155**(8):700-09.<br><br>Gafarov VV, Panov DO, Gromova EA, et al. The influence of social support on risk of acute cardiovascular diseases in female population aged 25-64 in Russia. International journal of circumpolar health 2013;**72**:21210.<br><br>Kang SH, Bloom JR. Social support and cancer screening among older black Americans. *Journal of the National Cancer Institute* 1993;**85**(j9j, 7503089):737-42.<br><br>Kawachi I, Colditz GA, Ascherio A, et al. A prospective study of social networks in relation to total mortality and cardiovascular disease in men in the USA. *Journal of Epidemiology and Community Health* 1996;**50**(3):245-51<br><br>Parboosingh EJ, Larsen DE. Factors Influencing Frequency and Appropriateness of Utilization of the Emergency Room by the Elderly. *Medical Care* 1987;**25**(12):1139-47.<br><br>Sykes D, Arveiler D, Salters CP, et al. Psychosocial risk factors for heart disease in France and Northern Ireland: the Prospective Epidemiological Study of Myocardial Infarction (PRIME). *International Journal of Epidemiology* 2002;**31**(6):1227-34.
11-item de Jong Gierveld Loneliness Scale [2]	Iecovich E, Biderman A. Attendance in adult day care centers and its relation to loneliness among frail older adults. *International Psychogeriatrics* 2012;**24**(3):439-48.<br><br>Kempen GI, Suurmeijer TP. Factors influencing professional home care utilization among the elderly. *Social Science & Medicine* 1991;**32**(1):77-81.
35-item Duke Social Support Index [3]	Hastings SN, George LK, Fillenbaum GG, Park RS, Burchett BM, Schmader KE. Does lack of social support lead to more ED visits for older adults? *The American Journal of Emergency Medicine* 2008;**26**(4):454-61.
11-item Duke Social Support Index [4]	Strodl E, Kenardy J, Aroney C. Perceived stress as a predictor of the self-reported new diagnosis of symptomatic CHD in older women. *International Journal of Behavioral Medicine* 2003;**10**(3):205-20.<br><br>Strodl E, Kenardy J. The 5-item mental health index predicts the initial diagnosis of nonfatal stroke in older women. *Journal of Women's Health* 2008;**17**(6):979-86.
4-item Duke Social Support Index [5]	Longman JM, I Rolfe M, Passey MD, et al. Frequent hospital admission of older people with chronic disease: a cross-sectional survey with telephone follow-up and data linkage. *BMC Health Services Research* 2012;**12**:373.
Duke-UNC Functional Social Support Questionnaire [6]	Ottenbacher KJ, Graham JE, Ottenbacher AJ, et al. Hospital readmission in persons with stroke following postacute inpatient rehabilitation. *The Journals of Gerontology: Series A, Biological Sciences and Medical Sciences* 2012;**67**(8): 875–881.<br><br>Stoddart H, Whitley E, Harvey I, Sharp D. What determines the use of home care services by elderly people? *Health & Social Care in the Community* 2002;**10**(5):348-60.
ENRICHD Social Support Inventory (ESSI) [7]	Coventry PA, Gemmell I, Todd CJ. Psychosocial risk factors for hospital readmission in COPD patients on early discharge services: A cohort study. *BMC Pulmonary Medicine* 2011;**11**.
Gijón Scale for the elderly's social-family assessment, family and social relationships subscales [8]	Lorén Guerrero L, Gascón Catalán M. Biopsychosocial factors related to the length of hospital stay in older people. *Revista Latino-Americana de Enfermagem* 2011;**19**(6):1377-84.
12-item Interpersonal Support Evaluation List (ISEL) [9]	Eakin EG, Strycker LA. Awareness and barriers to use of cancer support and information resources by HMO patients with breast, prostate, or colon cancer: patient and provider perspectives. *Psycho-oncology* 2001;**10**(2):103-13.
Interview Measure of Social Relationships [10]	Lesage AD, Charron M, Punti R, et al. Factors related to admission of new patients consulting geriatric psychiatric services in Montreal. *International Journal of Geriatric Psychiatry* 1994;**9**(8):663-72.
Litwin Support Network Types [11]	Litwin, H. Support network type and health service utilization. *Research on Aging* 1997;**19**:274–299.
10-item Lubben Social Network Scale [12]	Nagayoshi M, Everson-Rose SA, Iso H, Mosley TH, Rose KM, Lutsey PL. Social network, social support, and the risk of incident stroke: The atherosclerosis risk in communities study. *Circulation* 2014;**129**: 2868-73.<br><br>Player MS, King DE, Mainous AG, 3rd, Geesey ME. Psychosocial factors and progression from prehypertension to hypertension or coronary heart disease. *Annals of Family Medicine* 2007;**5**(5):403-11.

	Mistry R, Rosansky J, McGuire J, McDermott C, Jarvik L, Group UC. Social isolation predicts re-hospitalization in a group of older American veterans enrolled in the UPBEAT Program. *International Journal of Geriatric Psychiatry* 2001;**16**:950-959<br><br>Stoddart H, Whitley E, Harvey I, Sharp D. What determines the use of home care services by elderly people? *Health & Social Care in the Community* 2002;**10**(5):348-60.
6-item Lubben Social Network Scale [13]	Iliffe S, Kharicha K, Harari D, Swift C, Gillmann G, Stuck AE. Health risk appraisal in older people 2: The implications for clinicians and commissioners of social isolation risk in older people. *British Journal of General Practice* 2007;**57**(537):277-82.<br><br>Kobayashi KM, Cloutier-Fisher D, Roth M. Making meaningful connections: a profile of social isolation and health among older adults in small town and small city, British Columbia. *Journal of Aging and Health* 2009;**21**(2):374-97.<br><br>Nelms L, Johnson V, Teshuva K, Foreman P, Stanley J. Social and health factors affecting community service use by vulnerable older people. *Australian Social Work* 2009;**62**(4):507-24.<br><br>Simning A, Van Wijngaarden E, Fisher SG, Richardson TM, Conwell Y. Mental healthcare need and service utilization in older adults living in public housing. *American Journal of Geriatric Psychiatry* 2012;**20**(5):441-51.
Medical Outcomes Study (MOS) Social Support Survey [14]	de Boer AG, Sprangers MA, Speelman HD, de Haes HC. Predictors of health care use in patients with Parkinson's disease: a longitudinal study. *Movement Disorders* 1999;**14**(5):772-9
Multidimensional Scale of Perceived Social Support (MSPSS) [15]	Contrada RJ, Boulifard DA, Hekler EB, et al. Psychosocial factors in heart surgery: presurgical vulnerability and postsurgical recovery. *Health Psychology* 2008;**27**(3):309-19.<br><br>Simning A, Van Wijngaarden E, Fisher SG, Richardson TM, Conwell Y. Mental healthcare need and service utilization in older adults living in public housing. *American Journal of Geriatric Psychiatry* 2012;**20**(5):441-51.
Negative Affect Scale [16]	Burnette D, Mui AC. In-home and community-based service utilization by three groups of elderly Hispanics: a national perspective. *Social Work Research* 1995;**19**(4):197-206.<br><br>Tran TV, Dhooper SS, McInnis-Dittrich K. Utilization of community-based social and health services among foreign born Hispanic American elderly. *Journal of Gerontological Social Work* 1997;**28**(4):23-43.
Nottingham Health Profile Social Isolation subscale [17]	Naughton C, Drennan J, Treacy P, et al. How different are older people discharged from emergency departments compared with those admitted to hospital? *European Journal of Emergency Medicine* 2011;**18**(1):19-24.
Older Americans Research and Service Center (OARS) Social Resource Scale [18]	Almagro P, Barreiro B, De Echaguen AO, et al. Risk factors for hospital readmission in patients with chronic obstructive pulmonary disease. *Respiration* 2006;**73**(3):311-17.<br><br>Mcauley WJ, Arling G. Use of in-Home Care by Very Old-People. *Journal of Health and Social Behavior* 1984;**25**(1):54-64.<br><br>McCusker J, Healy E, Bellavance F, Connolly B. Predictors of repeat emergency department visits by elders. *Academic Emergency Medicine* 1997;**4**(6):581-88.
Oslo-3 Social Support Scale [19]	Boen H, Dalgard OS, Johansen R, Nord E. Socio-demographic, psychosocial and health characteristics of Norwegian senior centre users: a cross-sectional study. *Scandinavian Journal of Public Health* 2010;**38**(5):508-17.
Personal Resource Questionnaire (PRQ2000) [20]	Chen Y-J, Narsavage GL. Factors related to chronic obstructive pulmonary disease readmission in Taiwan. *Western Journal of Nursing Research* 2006;**28**(1):105-24.
University of California, Los Angeles (UCLA) Loneliness Scale [21]	[5-item version] Auslander GK, Soffer M, Auslander BA. The supportive community: Help seeking and service use among elderly people in Jerusalem. *Social Work Research* 2003;**27**(4):209-21.<br><br>[10-item version] Cheng ST. Loneliness-distress and physician utilization in well-elderly females. *Journal of Community Psychology* 1992;**20**(1):43-56.<br><br>[9-item version] Penning MJ. Health, social support, and the utilization of health services among older adults. *The Journals of Gerontology Series B: Psychological Sciences and Social Sciences* 1995; **50**(5):S330-9.
Wenger Support Network Typology [22]	Eustace A, Denihan A, Bruce I, Cunningham C, Coakley D, Lawlor BA. Depression in the community dwelling elderly: do clinical and sociodemographic factors influence referral to psychiatry? *International Journal of Geriatric Psychiatry* 2001;**16**(10):975-9.<br><br>Naughton C, Drennan J, Treacy P, et al. The role of health and non-health-related factors in repeat emergency department visits in an elderly urban population. *Emergency Medicine Journal* 2010;**27**(9):683-87.<br><br>Naughton C, Drennan J, Treacy P, et al. How different are older people discharged from emergency departments compared with those admitted to hospital? *European Journal of*

	Emergency Medicine 2011;**18**(1):19-24.
A measure of social isolation [23]	LaVeist TA, Sellers RM, Brown KA, Nickerson KJ. Extreme social isolation, use of community-based senior support services, and mortality among African American elderly women. *American Journal of Community Psychology* 1997; **25**(5):721-32
A measure of social network [24]	Mechakra-Tahiri SD, Zunzunegui MV, Dubé M, Préville M. Associations of social relationships with consultation for symptoms of depression: A community study of depression in older men and women in Québec. *Psychological Reports* 2011;**108**(2):537-52.
A measure of social anchorage [25]	Rennemark M, Holst G, Fagerstrom C, Halling A. Factors related to frequent usage of the primary healthcare services in old age: findings from The Swedish National Study on Aging and Care. *Health & Social Care in the Community* 2009;**17**(3):304-11.
Questionnaire on social network [26]	Rodriguez-Artalejo F, Guallar-Castillon P, Herrera MC, et al. Social network as a predictor of hospital readmission and mortality among older patients with heart failure. *Journal of Cardiac Failure 2006*;**12**(8):621-627.
Question about the number of sources of support [27]	Tennstedt SL, Crawford S, McKinlay JB. Determining the pattern of community care: is coresidence more important than caregiver relationship? *Journals of Gerontology: Social Sciences* 1993; **48**(2):S74-83.
An index of social support [28]	Lai DW. Use of home care services by elderly Chinese immigrants. *Home Health Care Services Quarterly* 2004;**23**(3):41-56.<br><br>Lai DWL, Kalyniak S. Use of annual physical examinations by aging Chinese Canadians. *Journal of Aging and Health* 2005;**17**(5):573-91.<br><br>Lai DWL. Predictors of use of senior centers by elderly Chinese immigrants in Canada. *Journal of Ethnic & Cultural Diversity in Social Work: Innovation in Theory, Research & Practice* 2006;**15**(12):97-121.
A measure of living arrangements and informal care [29]	Crets S. Determinants of the use of ambulant social care by the elderly. *Social Science & Medicine* 1996; **43**(12):1709-20.
A measure of satisfaction with social support [30]	Feld S, George LK. Moderating effects of prior social resources on the hospitalizations of elders who become widowed. *Journal of Aging and Health* 1994;**6**:275-95.
A measure of social integration [31]	Orth-Gomer K, Rosengren A, Wilhelmsen L. Lack of social support and incidence of coronary heart disease in middle-aged Swedish men. *Psychosomatic Medicine* 1993;**55**(1):37-43.<br><br>Rosengren A, Wilhelmsen L, Orth-Gomer K. Coronary disease in relation to social support and social class in Swedish men. A 15 year follow-up in the study of men born in 1933. *European Heart Journal* 2004;**25**(1):56-63.
A measure of social isolation [32]	Cloutier-Fisher D, Kobayashi KM. Examining social isolation by gender and geography: conceptual and operational challenges using population health data in Canada. *Gender Place and Culture* 2009;**16**(2):181-99.
A measure of social network [33]	Reed D, McGee D, Yano K, Feinleib M. Social networks and coronary heart disease among Japanese men in Hawaii. *American Journal of Epidemiology* 1983;**117**(4):384-96.
A measure of social network [34]	Reed D, McGee D, Yano K. Psychosocial processes and general susceptibility to chronic disease. *American Journal of Epidemiology* 1984;**119**(3):356-70.
A measure of social support [35]	Tran TV, Dhooper SS, McInnis-Dittrich K. Utilization of community-based social and health services among foreign born Hispanic American elderly. *Journal of Gerontological Social Work* 1997;**28**(4):23-43.
A measure of social support [36]	Andre-Petersson L, Hedblad B, Janzon L, Ostergren PO. Social support and behavior in a stressful situation in relation to myocardial infarction and mortality: who is at risk? Results from prospective cohort study "Men born in 1914," Malmo, Sweden. *International Journal of Behavioral Medicine* 2006;**13**(4):340-7.
A measure of social support [37]	Ikeda A, Iso H, Kawachi I, et al. Social support and stroke and coronary heart disease: the JPHC study cohorts II. *Stroke* 2008;**39**(3):768-75.
A measure of social support [38]	Kuper H, Adami HO, Theorell T, Weiderpass E. Psychosocial determinants of coronary heart disease in middle-aged women: a prospective study in Sweden. *American Journal of Epidemiology* 2006;**164**(4):349-57.
An social network index [39]	Rutledge T, Linke SE, Olson MB, et al. Social networks and incident stroke among women with suspected myocardial ischemia. *Psychosomatic Medicine* 2008;**70**(3):282-7.
Social network type [40]	Coe RM, Wolinsky FD, Miller DK, Prendergast JM. Social network relationships and use of physician services. A reexamination. *Research on Aging* 1984;**6**(2):243-56. Coe RM,<br><br>Coe RM, Wolinsky FD, Miller DK, Prendergast JM. Complementary and compensatory functions in social network relationships among the elderly. *Gerontologist* 1984;**24**(4):396-400<br><br>Wolinsky FD, Miller DK, Prendergast JM. Social network relationships and use of physician services. A reexamination. *Research on Aging* 1984;**6**():243-56.
Social network type – family [41]	Coe RM, Wolinsky FD, Miller DK, Prendergast JM. Elderly persons without family support networks and use of health services. A follow-up report on social network relationships. *Research on Aging* 1985;**7**(4):617-22.

Multi-item measures combining questions about frequency of contact with others and participation in activities, e.g. 'Over the last month were you able to: 1) attend events out-side of your home (e.g., community or social event); 2) visit friends or family in their own home?' [42]	John R, Roy LC, Dietz TL. Setting priorities in aging populations: formal service use among Mexican American female elders. *Journal of Aging & Social Policy* 1997;**9**(1):69-85.<br><br>Krout JA, Cutler SJ, Coward RT. Correlates of Senior Center Participation - a National Analysis. *Gerontologist* 1990;**30**(1):72-79.<br><br>Molloy GJ, McGee HM, O'Neill D, Conroy RM. Loneliness and emergency and planned hospitalizations in a community sample of older adults. *Journal of the American Geriatrics Society* 2010. **58**(8):1538-41.<br><br>Stump TE, Johnson RJ, Wolinsky FD. Changes in physician utilization over time among older adults. *The Journals of Gerontology Series B: Psychological Sciences and Social Sciences* 1995;**50**(1):S45-S58.<br><br>Wolinsky FD, Johnson RJ. The use of health services by older adults. *Journal of Gerontology* 1991;**46**(6):S345-57.
Question(s) about frequency of face to face and/or phone contact with family and/or friends and/or neighbours, e.g.: 'How many times during the past week did you spend some time with someone who does not live with you?' [43]	Aliyu MH, Adediran AS, Obisesan TO. Predictors of hospital admissions in the elderly: Analysis of data from the longitudinal study on aging. *Journal of the National Medical Association* 2003;**95**(12):1158-67.<br><br>Almind G, Holstein BE, Holst E, Due P. Old persons' contact with general practitioners in relation to health: a Danish population study. *Scandinavian Journal of Primary Health Care* 1991;**9**(4):252-8.<br><br>Auslander GK, Soffer M, Auslander BA. The supportive community: Help seeking and service use among elderly people in Jerusalem. *Social Work Research* 2003;**27**(4):209-21.<br><br>Barefoot JC, Gronbaek M, Jensen G, Schnohr P, Prescott E. Social network diversity and risks of ischemic heart disease and total mortality: findings from the Copenhagen City Heart Study. *American Journal of Epidemiology* 2005;**161**(10):960-7.<br><br>Burnette D, Mui AC. In-home and community-based service utilization by three groups of elderly Hispanics: a national perspective. *Social Work Research* 1995;**19**(4):197-206.<br><br>Burnette D, Mui AC. Physician utilization by Hispanic elderly persons - National perspective. *Medical Care* 1999;**37**(4):362-74.<br><br>Callahan CM, Wolinsky FD. Hospitalization for major depression among older Americans. *Journals of Gerontology - Series A Biological Sciences and Medical Sciences* 1995;**50**(4):M196-202.<br><br>Calsyn RJ, Winter JP. Who attends senior centers? *Journal of Social Service Research* 1999;**26**(2):53-69.<br><br>Chappell NL. Social support and the receipt of home care services. *Gerontologist* 1985;**25**(1):47-54.<br><br>Chappell NL, Blandford AA. Health-Service Utilization by Elderly Persons. *Canadian Journal of Sociology* 1987;**12**(3):195-215.<br><br>Choi NG. Patterns and determinants of social service utilization: comparison of the childless elderly and elderly parents living with or apart from their children. *Gerontologist* 1994;**34**(3):353-62.<br><br>Choi NG, Wodarski JS. The relationship between social support and health status of elderly people: does social support slow down physical and functional deterioration? *Social Work Research* 1996;**20**(1):52-63.<br><br>Crets S. Determinants of the use of ambulant social care by the elderly. *Social Science & Medicine* 1996; **43**(12):1709-20.<br><br>Dabelko HI, Balaswamy S. Use of adult day services and home health care services by older adults: a comparative analysis. *Home Health Care Services Quarterly* 2000;**18**(3):65-79.<br><br>Feld S, George LK. Moderating effects of prior social resources on the hospitalizations of elders who become widowed. *Journal of Aging and Health* 1994;**6**(3)275-95.<br><br>Frederiks CMA, Wierik MJM, Van Rossum HJL. Factors associated with differential utilization of professional care among elderly people: Residents of old people's homes compared to elderly people living at home. *Acta Hospitalia* 1991;**31**(3):33-45.<br><br>Ginsberg G, Israeli A, Cohen A, Stessman J. Factors predicting emergency room

utilization in a 70-year-old population. *Israel Journal of Medical Sciences* 1996;**32**(8):649-64.

Hedblad B, Ostergren PO, Hanson BS, Janzon L, Johansson BW, Juul-Moller S. Influence of social support on cardiac event rate in men with ischaemic type ST segment depression during ambulatory 24-h long-term ECG recording. *European Heart Journal* 1992;**13**(4):433-9.

Hyduk CA. The dynamic relationship between social support and health in older adults: Assessment implications. *Journal of Gerontological Social Work* 1996;**27**(1-2):149-65.

Iecovich E, Carmel S. Differences between users and nonusers of day care centers among frail older persons in Israel. *Journal of Applied Gerontology* 2011;**30**(4):443-62.

Jackson SA, Shiferaw B, Anderson RT, Heuser MD, Hutchinson KM, Mittelmark MB. Racial differences in service utilization: the Forsyth County Aging Study. *Journal of Health Care for the Poor & Underserved* 2002;**13**(3):320-33.

Jordan RE, Hawker JI, Ayres JG, et al. Effect of social factors on winter hospital admission for respiratory disease: a case-control study of older people in the UK. *The British Journal of General Practice* 2008;**58**(551):400-2.

Laditka JN, Laditka SB. Increased hospitalization risk for recently widowed older women and protective effects of social contacts. *Journal of Women & Aging* 2003;**15**(2-3):7-7.

Larsson K, Thorslund M, Forsell Y. Dementia and depressive symptoms as predictors of home help utilization among the oldest old: population-based study in an urban area of Sweden. *Journal of Aging and Health* 2004;**16**(5):641-68.

Miltiades HB, Wu B. Factors affecting physician visits in Chinese and Chinese immigrant samples. *Social Science & Medicine* 2008;**66**:704-714.
Nelson MA. Race, gender, and the effects of social supports on the use of health services by elderly individuals. *The International Journal of Aging & Human Development* 1993;**37**(3):227-46.

Ralston PA. Senior center utilization by black elderly adults: social, attitudinal and knowledge correlates. *Journal of Gerontology* 1984; **39**(2):224-9.
Redondo-Sendino Á, Guallar-Castillón P, Banegas JR, Rodríguez-Artalejo F. Gender differences in the utilization of health-care services among the older adult population of Spain. *BMC Public Health* 2006;**6**.

Rittner B, Kirk AB. Health care and public transportation use by poor and frail elderly people. *Social Work* 1995;**40**(3):365-73.

Solomon DH, Wagner DR, Marenberg ME, Acampora D, Cooney LM, Jr., Inouye SK. Predictors of formal home health care use in elderly patients after hospitalization. *Journal of the American Geriatrics Society* 1993; **41**(9):961-6.

Starrett RA, Todd AM, Deleon L. A Comparison of the Social-Service Utilization Behavior of the Cuban and Puerto-Rican Elderly. *Hispanic Journal of Behavioral Sciences* 1989;**11**(4):341-53.

Starrett RA, Decker JT, Araujo A, Walters G. The Cuban elderly and their service use. *Journal of Applied Gerontology* 1989;**8**(1):69-85.

Starrett RA, Bresler C, Decker JT, Walters GT, Rogers D. The role of environmental awareness and support networks in Hispanic elderly persons' use of formal social services. *Journal of Community Psychology* 1990;**18**(3):218-27.

Stump TE, Johnson RJ, Wolinsky FD. Changes in physician utilization over time among older adults. *The Journals of Gerontology Series B: Psychological Sciences and Social Sciences* 1995;**50**(1):S45-S58.

Thambypillai V. Utilization of formal social support services by non-institutionalized ill elderly. *Singapore Medical Journal* 1986;**27**(4):281-87.

Vogt TM, Mullooly JP, Ernst D, Pope CR, Hollis JF. Social networks as predictors of ischemic heart disease, cancer, stroke and hypertension: incidence, survival and mortality. *Journal of Clinical Epidemiology* 1992;**45**(6):659-66.

Walter-Ginzburg A, Chetrit A, Medina C, Blumstein T, Gindin J, Modan B. Physician visits, emergency room utilization, and overnight hospitalization in the old-old in Israel: The Cross-Sectional and Longitudinal Aging Study (CALAS). *Journal of the American Geriatrics Society* 2001;**49**(5):549-56.

	West GE, Delisle MA, Simard C, Drouin D. Leisure activities and service knowledge and use among the rural elderly. *Journal of Aging and Health* 1996; **8**(2):254-79.<br><br>Wilkins K, Beaudet MP. Changes in social support in relation to seniors' use of home care. *Health Reports* 2000; **11**(4):39-47.<br><br>Wolinsky FD, Johnson RJ. The use of health services by older adults. *Journal of Gerontology* 1991;46(6):S345-57.
Question(s) about the geographical proximity of family and friends [44]	Nagga K, Dong HJ, Marcusson J, Skoglund SO, Wressle E. Health-related factors associated with hospitalization for old people: Comparisons of elderly aged 85 in a population cohort study. *Archives of Gerontology and Geriatrics* 2012;**54**(2):391-397.
Question(s) about the number of close friends or relatives, e.g. asking respondents for the 'number of friends [they] feel close to' [45]	Chappell NL. Social support and the receipt of home care services. *Gerontologist* 1985;**25**(1):47-54.<br><br>Feld S, George LK. Moderating effects of prior social resources on the hospitalizations of elders who become widowed. *Journal of Aging and Health* 1994;**6**:275-95.<br><br>Lee BW, Conwell Y, Shah MN, Barker WH, Delavan RL, Friedman B. Major depression and emergency medical services utilization in community-dwelling elderly persons with disabilities. *International Journal of Geriatric Psychiatry* 2008;**23**(12):1276-82.
Question(s) about participation in social activities such as going to the cinema, sport events, church attendance or volunteering, e.g. 'In the past two weeks, did you go to a show or movie, sports event, club meeting, classes or other group event?' [46]	Aliyu MH, Adediran AS, Obisesan TO. Predictors of hospital admissions in the elderly: Analysis of data from the longitudinal study on aging. *Journal of the National Medical Association* 2003;**95**(12):1158-67.<br><br>Auslander GK, Soffer M, Auslander BA. The supportive community: Help seeking and service use among elderly people in Jerusalem. *Social Work Research* 2003;**27**(4):209-21.<br><br>Mechakra-Tahiri SD, Zunzunegui MV, Dubé M, Préville M. Associations of social relationships with consultation for symptoms of depression: A community study of depression in older men and women in Québec. *Psychological Reports* 2011;**108**(2):537-52.<br><br>Starrett RA, Todd AM, Deleon L. A Comparison of the Social-Service Utilization Behavior of the Cuban and Puerto-Rican Elderly. *Hispanic Journal of Behavioral Sciences* 1989;**11**(4):341-53.<br><br>Starrett RA, Decker JT, Araujo A, Walters G. The Cuban elderly and their service use. *Journal of Applied Gerontology* 1989;**8**(1):69-85.<br><br>Starrett RA, Bresler C, Decker JT, Walters GT, Rogers D. The role of environmental awareness and support networks in Hispanic elderly persons' use of formal social services. *Journal of Community Psychology* 1990;**18**(3):218-27.
Question(s) about the perceived availability of emotional, tangible, informational and/or other support, e.g. 'Is there someone who would give you any help at all if you were sick or disabled, for example your husband/wife, a member of your family, or a friend?' [47]	Alkema GE, Reyes JY, Wilber KH. Characteristics associated with home- and community-based service utilization for Medicare managed care consumers. *Gerontologist* 2006;**46**(2):173-82<br><br>Barresi CM, McConnell DJ. Adult day care participation among impaired elderly. *Lifestyles* 1987;**8**(3-4):82-94.<br><br>Black BS, Rabins PV, German P, McGuire M, Roca R. Need and unmet need for mental health care among elderly public housing residents. *Gerontologist* 1997;**37**(6):717-28.<br><br>Clay OJ, Roth DL, Safford MM, Sawyer PL, Allman RM. Predictors of Overnight Hospital Admission in Older African American and Caucasian Medicare Beneficiaries. *Journals of Gerontology Series a-Biological Sciences and Medical Sciences* 2011;**66**(8):910-16.<br><br>Ginsberg G, Israeli A, Cohen A, Stessman J. Factors predicting emergency room utilization in a 70-year-old population. *Israel Journal of Medical Sciences* 1996;**32**(8):649-64.<br><br>Hyduk CA. The dynamic relationship between social support and health in older adults: Assessment implications. *Journal of Gerontological Social Work* 1996;**27**(1-2):149-65.<br><br>Jackson SA, Shiferaw B, Anderson RT, Heuser MD, Hutchinson KM, Mittelmark MB. Racial differences in service utilization: the Forsyth County Aging Study. *Journal of Health Care for the Poor & Underserved* 2002;**13**(3):320-33.<br><br>Korten AE, Jacomb PA, Jiao Z, et al. Predictors of GP service use: a community survey of an elderly Australian sample. *Australian and New Zealand Journal of Public Health* 1998;**22**(5):609-15.<br><br>Krause N. Close companions at church, health, and health care use in late life. *Journal*

	of Aging and Health 2010;**22**(4):434-53.<br><br>Mechakra-Tahiri SD, Zunzunegui MV, Dubé M, Préville M. Associations of social relationships with consultation for symptoms of depression: A community study of depression in older men and women in Québec. *Psychological Reports* 2011;**108**(2):537-52.<br><br>Molloy GJ, McGee HM, O'Neill D, Conroy RM. Loneliness and emergency and planned hospitalizations in a community sample of older adults. *Journal of the American Geriatrics Society* 2010. **58**(8):1538-41.<br><br>Preville M, Vasiliadis HM, Boyer R, et al. Use of health services for psychological distress symptoms among community-dwelling older adults. *Canadian Journal on Aging* 2009;**28**(1):51-61.<br><br>Rittner B, Kirk AB. Health care and public transportation use by poor and frail elderly people. *Social Work* 1995;**40**(3):365-73.<br><br>Wan TT. Functionally disabled elderly. Health status, social support, and use of health services. *Research on Aging* 1987;**9**(1):61-78.<br><br>Wilkins K, Beaudet MP. Changes in social support in relation to seniors' use of home care. *Health Reports* 2000; **11**(4):39-47.
Question(s) about received support, e.g. asking participants whether they received assistance during the past month with 7 tasks, including shopping, housework or going to the doctor [48]	Auslander GK, Soffer M, Auslander BA. The supportive community: Help seeking and service use among elderly people in Jerusalem. *Social Work Research* 2003;**27**(4):209-21.<br><br>Bazargan M, Bazargan S, Baker RS. Emergency department utilization, hospital admissions, and physician visits among elderly African American persons. *Gerontologist* 1998;**38**(1):25-36.<br><br>Chappell NL, Blandford AA. Health-Service Utilization by Elderly Persons. *Canadian Journal of Sociology* 1987;**12**(3):195-215.<br><br>Cho E. The effects of nonprofessional caregivers on the rehospitalization of elderly recipients in home healthcare. *Advances in Nursing Science* 2007;**30**(3):E1-12.<br><br>Choi NG, Wodarski JS. The relationship between social support and health status of elderly people: does social support slow down physical and functional deterioration? *Social Work Research* 1996;**20**(1):52-63.<br><br>Crets S. Determinants of the use of ambulant social care by the elderly. *Social Science & Medicine* 1996; **43**(12):1709-20.<br><br>Frederiks CMA, Wierik MJM, Van Rossum HJL. Factors associated with differential utilization of professional care among elderly people: Residents of old people's homes compared to elderly people living at home. *Acta Hospitalia* 1991;**31**(3):33-45.<br><br>Hakim EA, Bakheit AM. A study of the factors which influence the length of hospital stay of stroke patients. *Clinical Rehabilitation* 1998;**12**(2):151-6.<br><br>Houde SC. Predictors of elders' and family caregivers' use of formal home services. *Research in Nursing & Health* 1998;**21**(6):533-43.<br><br>Jackson SA, Shiferaw B, Anderson RT, Heuser MD, Hutchinson KM, Mittelmark MB. Racial differences in service utilization: the Forsyth County Aging Study. *Journal of Health Care for the Poor & Underserved* 2002;**13**(3):320-33.<br><br>John R, Roy LC, Dietz TL. Setting priorities in aging populations: formal service use among Mexican American female elders. *Journal of Aging & Social Policy* 1997;**9**(1):69-85.<br><br>Kim H-S, Miyashita M, Harada K, Park J-H, So J-M, Nakamura Y. Psychological, social, and environmental factors associated with utilization of senior centers among older adults in Korea. *Journal of Preventive Medicine and Public Health* 2012;**45**(4):244-50<br><br>Larsson K, Thorslund M, Forsell Y. Dementia and depressive symptoms as predictors of home help utilization among the oldest old: population-based study in an urban area of Sweden. *Journal of Aging and Health* 2004;**16**(5):641-68.<br><br>Li LW. Caregiving Network Compositions and Use of Supportive Services by Community-Dwelling Dependent Elders. *Journal of Gerontological Social Work* 2004;**43**(2-3):147-64.<br><br>Miner S. Racial differences in family support and formal service utilization among older

	persons: A nonrecursive model. *The Journals of Gerontology Series B: Psychological Sciences and Social Sciences* 1995;**50**(3):S143-53.<br><br>Nelms L, Johnson V, Teshuva K, Foreman P, Stanley J. Social and health factors affecting community service use by vulnerable older people. *Australian Social Work* 2009;**62**(4):507-24.<br><br>Penning MJ. Health, social support, and the utilization of health services among older adults. *The Journals of Gerontology Series B: Psychological Sciences and Social Sciences* 1995; **50**(5):S330-9.<br><br>Safran DG, Graham JD, Osberg JS. Social supports as a determinant of community-based care utilization among rehabilitation patients. *Health Services Research* 1994;**28**(6):729-50<br><br>Smith GC. Patterns and predictors of service use and unmet needs among aging families of adults with severe mental illness. *Psychiatric Services* 2003;**54**(6):871-7.<br><br>Tomassini C, Glaser K, Stuchbury R. Family disruption and support in later life: A comparative study between the United Kingdom and Italy. *Journal of Social Issues* 2007;**63**(4):845-63.
Question(s) about satisfaction with social relationships and/or participation, e.g. asking participants whether they believe their present level of social activities to be adequate [49]	Auslander GK, Soffer M, Auslander BA. The supportive community: Help seeking and service use among elderly people in Jerusalem. *Social Work Research* 2003;**27**(4):209-21.<br><br>Blalock SJ, Byrd JE, Hansen RA, et al. Factors associated with potentially inappropriate drug utilization in a sample of rural community-dwelling older adults. *American Journal Geriatric Pharmacotherapy* 2005;**3**(3):168-79.<br><br>Boen H, Dalgard OS, Johansen R, Nord E. Socio-demographic, psychosocial and health characteristics of Norwegian senior centre users: a cross-sectional study. *Scandinavian Journal of Public Health* 2010;**38**(5):508-17.<br><br>Callahan CM, Wolinsky FD. Hospitalization for major depression among older Americans. *Journals of Gerontology - Series A Biological Sciences and Medical Sciences* 1995;**50**(4):M196-202.<br><br>Chappell NL. Social support and the receipt of home care services. *Gerontologist* 1985;**25**(1):47-54.<br><br>Conforti DA, Basic D, Rowland JT. Emergency department admissions, older people, functional decline, and length of stay in hospital. *Australasian Journal on Ageing* 2004;**23**(4):189-94.
Question(s) about the size of a person's network , e.g. number of friends and relatives outside the household [50]	Chappell NL. Social support and the receipt of home care services. *Gerontologist* 1985;**25**(1):47-54.<br><br>Chappell NL, Blandford AA. Health-Service Utilization by Elderly Persons. *Canadian Journal of Sociology* 1987;**12**(3):195-215.<br><br>Feld S, George LK. Moderating effects of prior social resources on the hospitalizations of elders who become widowed. *Journal of Aging and Health* 1994;**6**:275-95.<br><br>Vogt TM, Mullooly JP, Ernst D, Pope CR, Hollis JF. Social networks as predictors of ischemic heart disease, cancer, stroke and hypertension: incidence, survival and mortality. *Journal of Clinical Epidemiology* 1992;**45**(6):659-66.<br><br>Wan TT. Functionally disabled elderly. Health status, social support, and use of health services. *Research on Aging* 1987;**9**(1):61-78.
Question about time spent alone [51]	Ginsberg G, Israeli A, Cohen A, Stessman J. Factors predicting emergency room utilization in a 70-year-old population. *Israel Journal of Medical Sciences* 1996;**32**(8):649-64.
Single-item question about feeling lonely, e.g.: 'How often in the last 12 months have you been bothered by loneliness?' [42]	Almind G, Holstein BE, Holst E, Due P. Old persons' contact with general practitioners in relation to health: a Danish population study. *Scandinavian Journal of Primary Health Care* 1991;**9**(4):252-8.<br><br>Calsyn RJ, Winter JP. Who attends senior centers? *Journal of Social Service Research* 1999;**26**(2):53-69.<br><br>Eaker ED, Pinsky J, Castelli WP. Myocardial infarction and coronary death among women: psychosocial predictors from a 20-year follow-up of women in the Framingham Study. *American Journal of Epidemiology* 1992;**135**(8):854-64.<br><br>Frederiks CMA, Wierik MJM, Van Rossum HJL. Factors associated with differential utilization of professional care among elderly people: Residents of old people's homes

	compared to elderly people living at home. *Acta Hospitalia* 1991;**31**(3):33-45.
	Ginsberg G, Israeli A, Cohen A, Stessman J. Factors predicting emergency room utilization in a 70-year-old population. *Israel Journal of Medical Sciences* 1996;**32**(8):649-64.
	Lofvenmark C, Mattiasson A-C, Billing E, Edner M. Perceived loneliness and social support in patients with chronic heart failure. *European Journal of Cardiovascular Nursing* 2009;**8**(4):251-8.
	Molloy GJ, McGee HM, O'Neill D, Conroy RM. Loneliness and emergency and planned hospitalizations in a community sample of older adults. *Journal of the American Geriatrics Society* 2010. **58**(8):1538-41.
	Nagga K, Dong HJ, Marcusson J, Skoglund SO, Wressle E. Health-related factors associated with hospitalization for old people: Comparisons of elderly aged 85 in a population cohort study. *Archives of Gerontology and Geriatrics* 2012;**54**(2):391-397.
	Thurston RC, Kubzansky LD. Women, loneliness, and incident coronary heart disease. *Psychosomatic Medicine* 2009;**71**(8):836-42.

References

1. Berkman LF, Breslow L. *Health and ways of living*. New York: Oxford University Press, 1983.
2. de Jong Gierveld J, van Tilburg T. A 6-item scale for overall, emotional, and social loneliness. *Research on Aging* 2006;**28**(5):582-98.
3. Landerman R, George LK, Campbell RT, Blazer DG. Alternative models of the stress buffering hypothesis. *American Journal of Community Psychology* 1989;**17**(5):625-42.
4. Powers JR, Goodger B, Byles JE. Assessment of the abbreviated Duke Social Support Index in a cohort of older Australian women. *Australasian Journal on Aging* 2004;**23**(2):71-76.
5. Longman JM, M IR, Passey MD, et al. Frequent hospital admission of older people with chronic disease: a cross-sectional survey with telephone follow-up and data linkage. *BMC Health Services Research* 2012;**12**:373.
6. Broadhead WE, Gehlbach SH, de Gruy FV, Kaplan BH. The Duke-UNC Functional Social Support Questionnaire. Measurement of social support in family medicine patients. *Medical Care* 1988;**26**(7):709-23.
7. Mitchell PH, Powell L, Blumenthal J, et al. A short social support measure for patients recovering from myocardial infarction: the ENRICHD Social Support Inventory. *Journal of Cardiopulmonary Rehabilitation* 2003;**23**(6):398-403.
8. García-González JV, Díaz-Palacios E, Salamea A, et al. Evaluación de la fiabilidad y validez de una escala de valoración social en el anciano. *Atención Primaria* 1999;**23**(7):434-40.
9. Cohen S, Memelstein R, Kamarck T, Hoberman H. Measuring the functional components of social support. In: Sarason IG, Sarason B, eds. *Social support: Theory, research and application*. The Hague: Martinus Nijhoff, 1985:73-94.
10. Brugha TS, Sturt E, MacCarthy B, Potter J, Wykes T, Bebbington PE. The Interview Measure of Social Relationships: the description and evaluation of a survey instrument for assessing personal social resources. *Social Psychiatry* 1987;**22**(2):123-8.
11. Litwin H. Support Network Type and Health Service Utilization. *Research on Aging* 1997;**19**:274-99.
12. Lubben J. Assessing social networks among elderly populations. *Family and Community Health* 1988;**11**(3):42-52.
13. Lubben J, Blozik E, Gillmann G, et al. Performance of an abbreviated version of the Lubben Social Network Scale among three European community-dwelling older adult populations. *Gerontologist* 2006;**46**(4):503-13.

14. Sherbourne CD, Stewart AL. The MOS Social Support Survey. *Social Science & Medicine* 1991;**32**(713-714).
15. Zimet GD, Dahlem NW, Zimet SG, Farley GK. The Multidimensional Scale of Perceived Social Support. *Journal of Personality Assessment* 1988;**52**(1):30-41.
16. Bradburn NM. *The structure of psychological well-being*. Chicago: Aldine, 1969.
17. Hunt SM, McKenna SP, McEwen J, Williams J, Papp E. The Nottingham Health Profile: subjective health status and medical consultations. *Social Science & Medicine* 1981;**15**(3 Pt 1):221-9.
18. Fillenbaum GG. Multidimensional Functional Assessment of Older Adults: The Duke Older Americans Resources and Services Procedures, 1988.
19. Boen H, Dalgard OS, Johansen R, Nord E. Socio-demographic, psychosocial and health characteristics of Norwegian senior centre users: a cross-sectional study. *Scandinavian Journal of Public Health* 2010;**38**(5):508-17.
20. Weinert C. Evaluation of the Personal Resource Questionnaire: a social support measure. *Birth Defects Original Article Series* 1984;**20**(5):59-97.
21. Russell D, Peplau LA, Ferguson ML. Developing a measure of loneliness. *Journal of Personality Assessment* 1978;**42**(3):290-4.
22. Wenger GC. A network typology: From theory to practice. *Journal of Aging Studies* 1991;**5**(2):147-62.
23. LaVeist TA, Sellers RM, Brown KA, Nickerson KJ. Extreme social isolation, use of community-based senior support services, and mortality among African American elderly women. *American Journal of Community Psychology* 1997;**25**(5):721-32.
24. Mechakra-Tahiri SD, Zunzunegui MV, Dubé M, Préville M. Associations of social relationships with consultation for symptoms of depression: A community study of depression in older men and women in Québec. *Psychological Reports* 2011;**108**(2):537-52.
25. Rennemark M, Holst G, Fagerstrom C, Halling A. Factors related to frequent usage of the primary healthcare services in old age: findings from The Swedish National Study on Aging and Care. *Health & Social Care in the Community* 2009;**17**(3):304-11.
26. Rodriguez-Artalejo F, Guallar-Castillon P, Herrera MC, et al. Social network as a predictor of hospital readmission and mortality among older patients with heart failure. *Journal of Cardiac Failure* 2006;**12**(8):621-7.
27. Tennstedt SL, Crawford S, McKinlay JB. Determining the pattern of community care: is coresidence more important than caregiver relationship? *Journals of Gerontology: Social Sciences* 1993;**48**(2):S74-83.
28. Lai DWL. Predictors of Use of Senior Centers by Elderly Chinese Immigrants in Canada. *Journal of Ethnic and Cultural Diversity in Social Work* 2006;**15**(1-2):97-121.
29. Crets S. Determinants of the use of ambulant social care by the elderly. *Social Science & Medicine* 1996;**43**(12):1709-20.
30. Feld S, George LK. Moderating effects of prior social resources on the hospitalizations of elders who become widowed. *Journal of Aging and Health* 1994;**6**:275-95.
31. Orth-Gomer K, Rosengren A, Wilhelmsen L. Lack of social support and incidence of coronary heart disease in middle-aged Swedish men. *Psychosomatic Medicine* 1993;**55**(1):37-43.
32. Cloutier-Fisher D, Kobayashi KM. Examining social isolation by gender and geography: conceptual and operational challenges using population health data in Canada. *Gender Place and Culture* 2009;**16**(2):181-99.
33. Reed D, McGee D, Yano K, Feinleib M. Social networks and coronary heart disease among Japanese men in Hawaii. *American Journal of Epidemiology* 1983;**117**(4):384-96.
34. Reed D, McGee D, Yano K. Psychosocial processes and general susceptibility to chronic disease. *American Journal of Epidemiology* 1984;**119**(3):356-70.
35. Tran TV, Dhooper SS, McInnis-Dittrich K. Utilization of community-based social and health services among foreign born Hispanic American elderly. *Journal of Gerontological Social Work* 1997;**28**(4):23-43.

36. Andre-Petersson L, Hedblad B, Janzon L, Ostergren PO. Social support and behavior in a stressful situation in relation to myocardial infarction and mortality: who is at risk? Results from prospective cohort study "Men born in 1914," Malmo, Sweden. *International Journal of Behavioral Medicine* 2006;**13**(4):340-7.
37. Ikeda A, Iso H, Kawachi I, et al. Social support and stroke and coronary heart disease: the JPHC study cohorts II. *Stroke* 2008;**39**(3):768-75.
38. Kuper H, Adami HO, Theorell T, Weiderpass E. Psychosocial determinants of coronary heart disease in middle-aged women: a prospective study in Sweden. *American Journal of Epidemiology* 2006;**164**(4):349-57.
39. Rutledge T, Linke SE, Olson MB, et al. Social networks and incident stroke among women with suspected myocardial ischemia. *Psychosomatic Medicine* 2008;**70**(3):282-7.
40. Coe RM, Wolinsky FD, Miller DK, Prendergast JM. Social network relationships and use of physician services. A reexamination. *Research on Aging* 1984;**6**(2):243-56.
41. Coe RM, Wolinsky FD, Miller DK, Prendergast JM. Elderly persons without family support networks and use of health services. A follow-up report on social network relationships. *Research on Aging* 1985;**7**(4):617-22.
42. Molloy GJ, McGee HM, O'Neill D, Conroy RM. Loneliness and emergency and planned hospitalizations in a community sample of older adults. *Journal of the American Geriatrics Society* 2010;**58**(8):1538-41.
43. Hyduk CA. The dynamic relationship between social support and health in older adults: Assessment implications. *Journal of Gerontological Social Work* 1996;**27**(1-2):149-65.
44. Nagga K, Dong HJ, Marcusson J, Skoglund SO, Wressle E. Health-related factors associated with hospitalization for old people: Comparisons of elderly aged 85 in a population cohort study. *Archives of Gerontology and Geriatrics* 2012;**54**(2):391-97.
45. Lee BW, Conwell Y, Shah MN, Barker WH, Delavan RL, Friedman B. Major depression and emergency medical services utilization in community-dwelling elderly persons with disabilities. *International Journal of Geriatric Psychiatry* 2008;**23**(12):1276-82.
46. Kovar MG, Fitti JE, Chyba MM. The Longitudinal Study of Aging: 1984-90. *Vital Health Statistics, Series 1* 1992(28):1-248.
47. Barresi CM, McConnell DJ. Adult day care participation among impaired elderly. *Lifestyles* 1987;**8**(3-4):82-94.
48. Auslander GK, Soffer M, Auslander BA. The supportive community: Help seeking and service use among elderly people in Jerusalem. *Social Work Research* 2003;**27**(4):209-21.
49. Callahan CM, Wolinsky FD. Hospitalization for major depression among older Americans. *Journals of Gerontology - Series A Biological Sciences and Medical Sciences* 1995;**50**(4):M196-202.
50. Chappell NL, Blandford AA. Health-Service Utilization by Elderly Persons. *Canadian Journal of Sociology* 1987;**12**(3):195-215.
51. Ginsberg G, Israeli A, Cohen A, Stessman J. Factors predicting emergency room utilization in a 70-year-old population. *Israel Journal of Medical Sciences* 1996;**32**(8):649-64.
